# Tell or hide the truth from patients? The role of bioethics in medicine

**DOI:** 10.1051/sicotj/2025053

**Published:** 2025-09-24

**Authors:** Konstantinos V. Tsihrintzis, Maria Anthi Kouri, Ioannis M. Koukourakis, Panagoula Oikonomou, Andreas F. Mavrogenis

**Affiliations:** 1 Postgraduate Program “Bioethics”, Democritus University of Thrace, School of Medicine Alexandroupolis 68100 Greece; 2 Department of Applied Medical Physics, National and Kapodistrian University of Athens, School of Medicine Athens 12461 Greece; 3 Radiation Oncology Unit, National and Kapodistrian University of Athens, School of Medicine, Aretaieion Hospital Athens 11528 Greece; 4 Laboratory of Experimental Surgery, Democritus University of Thrace, School of Medicine Alexandroupolis 68100 Greece; 5 First Department of Orthopaedics, National and Kapodistrian University of Athens, School of Medicine Athens 12461 Greece

**Keywords:** Bioethics, Medical ethics, Physician-patient communication, Medical education, Orthopaedics

## Abstract

Physicians often grapple with the delicate balance between providing full disclosure and shielding patients from harsh realities. Honesty, empathy, and patient-centered care are crucial elements influencing patient outcomes and well-being. The revelation process of life-threatening diseases triggers distinct psychological coping stages, emphasizing the need for sensitive communication. Cultural factors further shape communication dynamics, necessitating individualized approaches. As such, this paper discusses the need for truth in the relationships and interactions of doctors and patients, emphasizes adequate information of patients based on honesty and consideration of their expectations, environment, and cultural values, and explores the pivotal role of bioethics education and training in preparing medical professionals to navigate these complex situations. By integrating bioethics education into medical curricula, fostering open and honest communication, and building strong patient-doctor relationships, we can enhance the quality of care and empower patients to embrace their medical journey with dignity and acceptance.

## Introduction

The doctor-patient relationship has been and remains a keystone of care. This relationship has received philosophical, sociological, and literary attention since Hippocrates. It is critical for vulnerable patients as they experience a heightened reliance on their physician’s competence, skills, and goodwill. For this relationship to be robust, it should focus on the truth that is the property of being in accord with fact or reality. The presence of truth constitutes a permanent demand in any form of relationship. Truth breeds trust. The first, perhaps, of all the tips and tricks that a doctor is taught in medical school is to make the patient his ally, to devote time to him, to gain his trust. But what about patients who are facing a chronic, progressive, or fatal disease? Fully communicating the diagnosis, outcome, therapy, and evolution of a specific disease or hiding the truth is best in these patients? In many cases, the primary urge pertains to the embellishment of the tough truth in an attempt to soften the inevitable reality. However, as Benjamin Franklin used to say, “…*Half a truth is often a great lie*…”. Therefore, how ethical is hiding the truth from patients?

## Interaction with patients – Anxiety and distress

The revelation process of a potentially fatal disease is commonly described as a short blunt announcement and is characterized by the certainty of the diagnosis and the uncertainty of the prognosis. There are three scenarios worldwide that the majority of physicians follow in order to interact with a patient with a life-threatening disease. According to the first scenario, the patient can only receive information regarding treatment procedures. The second scenario pertains to the full enlightenment of the patient. Finally, according to the third scenario, the extent of the information provided by the patients can be customized [1].

The interesting observation from the above interactions with patients is that the level of impact and thus the level of honesty may even affect the progress of the disease. Research has shown that the doctor’s competency in addressing emotional concerns and presenting empathy has been correlated to patients’ higher health-related quality of life [2]. Furthermore, full disclosure in patients’ informed consent to their disease can be correlated with attention, emotional support and eventually a patient-centered and facilitative approach to care [3], which could potentially lead to lower levels of anxiety and distress [4].

## Announcing the diagnosis – Denial, frustration, bargaining, and acceptance

Announcing an unpleasant diagnosis is the inevitable first step of the many complicated and painful steps that are expected to follow for the patients and their relatives. The mental processing of the diagnosis, whether based on what is explicitly known or internally feared, is closely linked to the existential fear of death. Typically, patients exhibit a multi-stage psychological reaction. The first stage is commonly characterized by denial and often accompanied by isolation. This is usually followed by anger, frustration, bargaining where patients attempt to negotiate or make deals in hopes of delaying or altering the course of their disease, and depression. Finally, acceptance may occur, although many patients may never fully reach this final stage [5–7].

Acceptance of the disease is vital. According to psychoanalytic theories, patients who can grasp the progression of their disease – its personal implications, limitations, or even the potential for death – are more likely to reconstruct their sense of identity, focus on improving quality of life, and move toward psychological acceptance [8, 9]. This transformative process is often facilitated when a trusted authority figure, such as a physician, guides the patient through it with clarity and empathy [8].

Honesty is a non-negotiable ethical cornerstone in this process. However, communicating such life-altering information is emotionally taxing for both the physician and the patient. For the patient, the shock often begins with the mere announcement of a terminal diagnosis. This emotional toll then escalates as the patient must process a flood of information, evaluate treatment options, and make urgent, often overwhelming decisions. As a result, many patients struggle to fully comprehend or emotionally accept the gravity of their situation.

## Considering patients’ expectations – Environment and cultures

**Figure 1:**
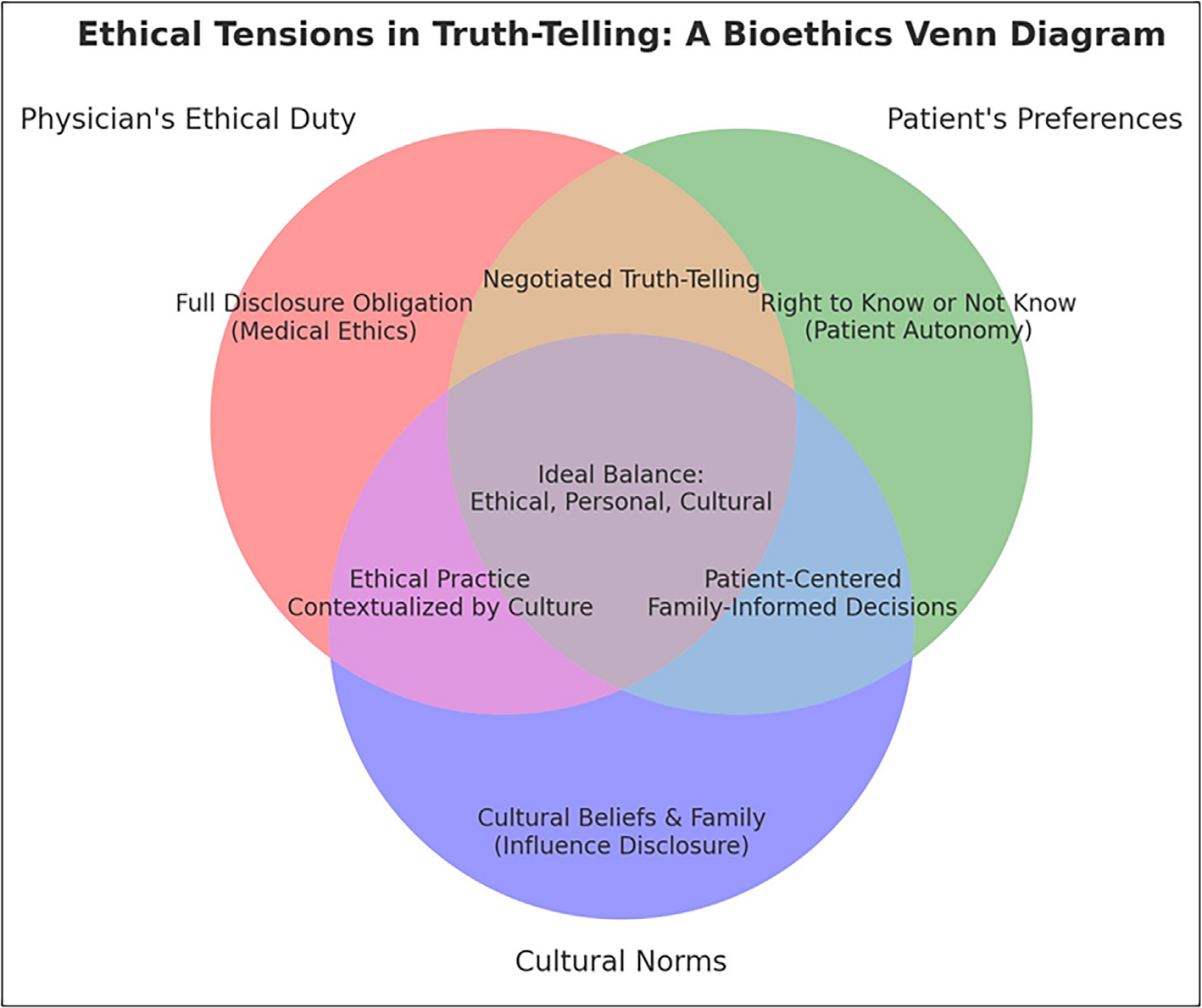
Visualization of the interplay between a physician’s ethical obligation to disclose medical truths, a patient’s right to know or not know, and the influence of cultural norms or family preferences. At their intersection lies the ideal: a communication approach that is ethically sound, patient-centered, and culturally sensitive. This model highlights the complexity of disclosure in practice and underscores the need for structured bioethics training to help clinicians navigate these overlapping domains.

Next, the values and preferences of the patients and their relatives should be given the highest priority. To better understand those values, physicians should consider the patients’ environment, coping mechanisms, level of stress, and psychological status. Importantly, it is vital to take into consideration any cross-cultural issues that may add variables to the patient’s perception. For example, in the Eastern world, the families play a significant role in the management of a patient member of the family; therefore, hiding the truth or revealing only a small part of it, is a common situation and explicit request on behalf of the family environment. Similarly, a paternalistic model extracts the patients’ needs and personality out from the picture. Moreover, the belief that the patients’ health will deteriorate after they are informed about their medical condition exists and is commonly adopted [9–11]. In contrast, in the Western world, patients act independently to ensure they get the “final say” regarding their treatment. In this culture, different strategies are considered a violation of basic human rights. Thus, the patients’ individual characteristics and personality have been taken severely into consideration and customarily determine any health update announcements. In other words, physicians provide them with as much of the truth as it is possible to handle [11]. These overlapping concerns often come into conflict: what the physician is ethically obligated to disclose; what the patient wishes to hear; and what the cultural context permits. The intersection of these three forces represents the ethical space in which physicians must operate (Figure 1). In any case, physicians owe it listen carefully to patients’ needs, as well as to act and advise them accordingly towards the direction of their health improvement [12].

There are very few papers on the relationship between clinicians and their patients [13–15]. The bedside manner refers to “the way in which a doctor treats people who are ill, especially showing kind, friendly, and understanding behaviour”. This description essentially describes how we would all like to be treated if and when we or our family members become patients. There is no point in having a brilliant mind but no mechanism for expressing it. Providing comfort to patients allows for a genuine two-way sharing of information, benefiting both parties [15].

## Informing orthopaedic patients – Physical recovery vs. return to work

The specialism of orthopaedic surgery covers many subdisciplines; most of these deal with degenerative changes occurring as a result of the normal aging process, some will follow trauma, others will result from congenital malformations, tumors, or infections [16–21]. Patients are pre-conditioned to look for a cure and are not that happy to learn if one is not available [22]. These patients need careful handling as they need to understand their disease, that some processes cannot be cured, and resultant symptoms can only be managed. Orthopaedic meetings are full of examples where there is a “triumph of technology over common sense”. It is apparent that orthopaedic patients are more interested in physical recovery than in a return to work or financial objectives. For that reason, orthopaedic surgeons should strongly consider assessing the socioeconomic well-being of their patients [22, 23].

**Figure 2:**
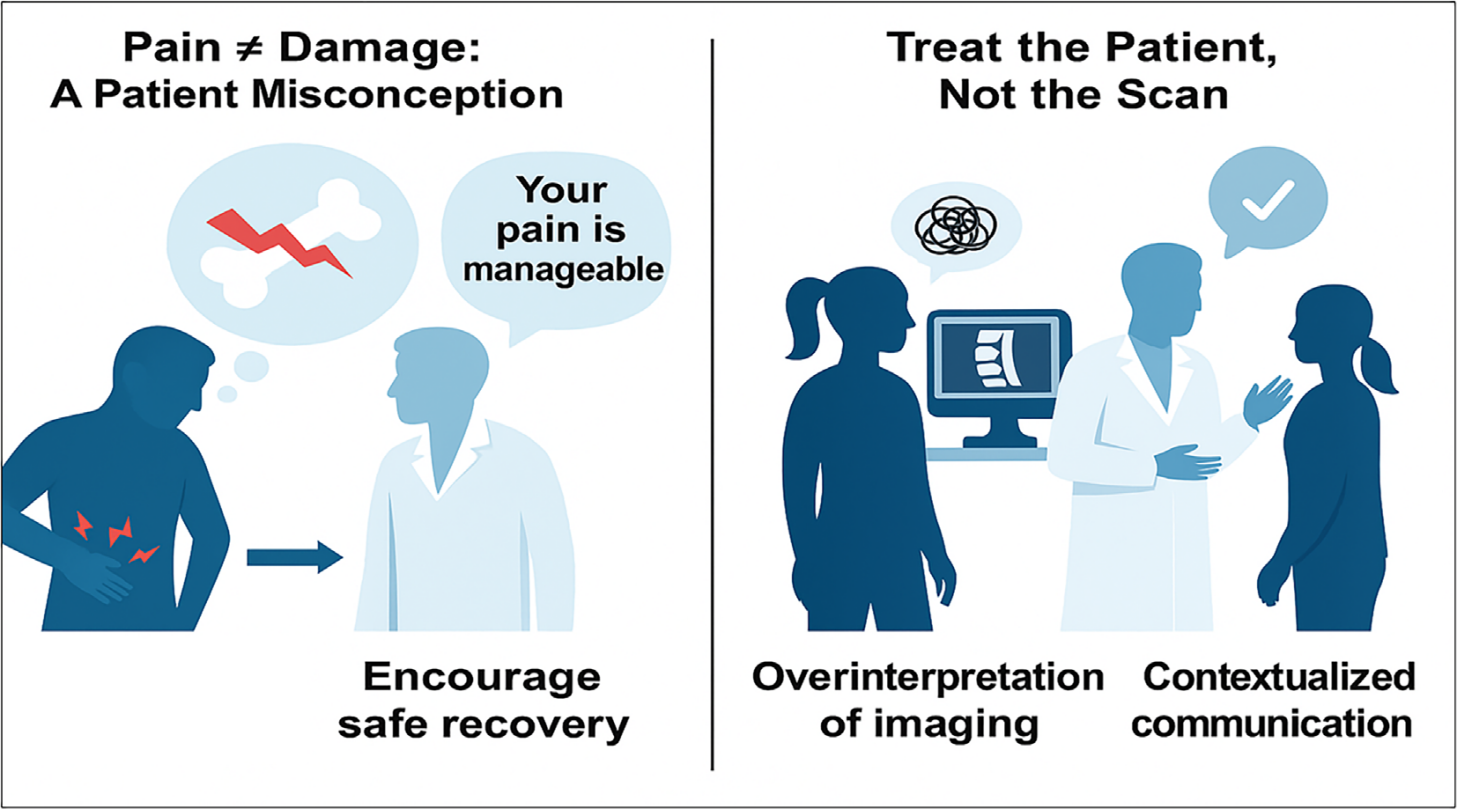
The two key communication challenges in orthopaedic care. On the left, patients often equate pain with serious injury, leading to avoidance and delayed recovery. On the right, overreliance on imaging may reinforce fear, whereas patient-centered explanation and reassurance can promote activity, reduce anxiety, and improve outcomes. These examples highlight the importance of clinical empathy, contextual interpretation, and patient education in the management of musculoskeletal conditions.

Pain is a personal experience. The way individuals react to pain is very different and very subjective. Most patients are convinced that the experience of pain means that they are suffering major damage, and they are concerned that any activity that causes pain should be avoided. They often need permission to return to activity, and delay their return to work for months. Misconceptions about pain, the overinterpretation of imaging results, and a lack of personalized communication contribute significantly to delayed recovery. Visual and verbal cues from clinicians can shape patient understanding of pain and diagnostic imaging (Figure 2). It is the responsibility of the treating physicians to reassure their patients that musculoskeletal symptoms are rarely the source of permanent injury; imaging investigations such as MRI scans have a valuable role in explaining and reassuring patients that there is no significant or important pathology that requires invasive treatment [22]. In this setting, caution should be given to treat the patient, not the scans. This situation is likely to become more acute with the rise of artificial intelligence, which can certainly improve diagnosis, but not the interaction with a patient.

With the challenges of more informed and demanding patients, physicians must improve and practice their interpersonal skills. Information for patients is certainly key, as an informed patient is a more understanding and cooperative patient, as they can understand the reasoning behind clinical decisions, and are more compliant with the proposed treatment [15].

## Comforting patients – Bioethics training and education

There is no perfect strategy or golden standard for comforting a patient with a critical health problem. In that distress condition, the physicians should have prepared their approach and be mindful of the tone of their voice, their selection of words, and their body language, while simultaneously maintaining their authenticity and comfort. They should demonstrate lucidity and sincerity and undertake humanistic and ethical reflection. Hesitation and ambiguity cause insecurity in patients and disruption of relationships. Telling the truth was never a simple task; it requires a balance of cultural and personal issues, without the patient’s inspiration and hope being lost [12]. It requires that the physician consider the physical, psychological, social, and health-related quality of life measures for each individual patient [3–5, 22, 24, 25]. Additionally, the surgeons should understand the adverse psychology of their patients and determine those patients who will not do well with a surgical procedure for a given disease. Moreover, patient-reported outcome measurements (PROMs) are problematic because depressive symptoms and catastrophising appear to be key factors influencing their score and the scoring of clinician-rated outcomes [26].

**Figure 3:**
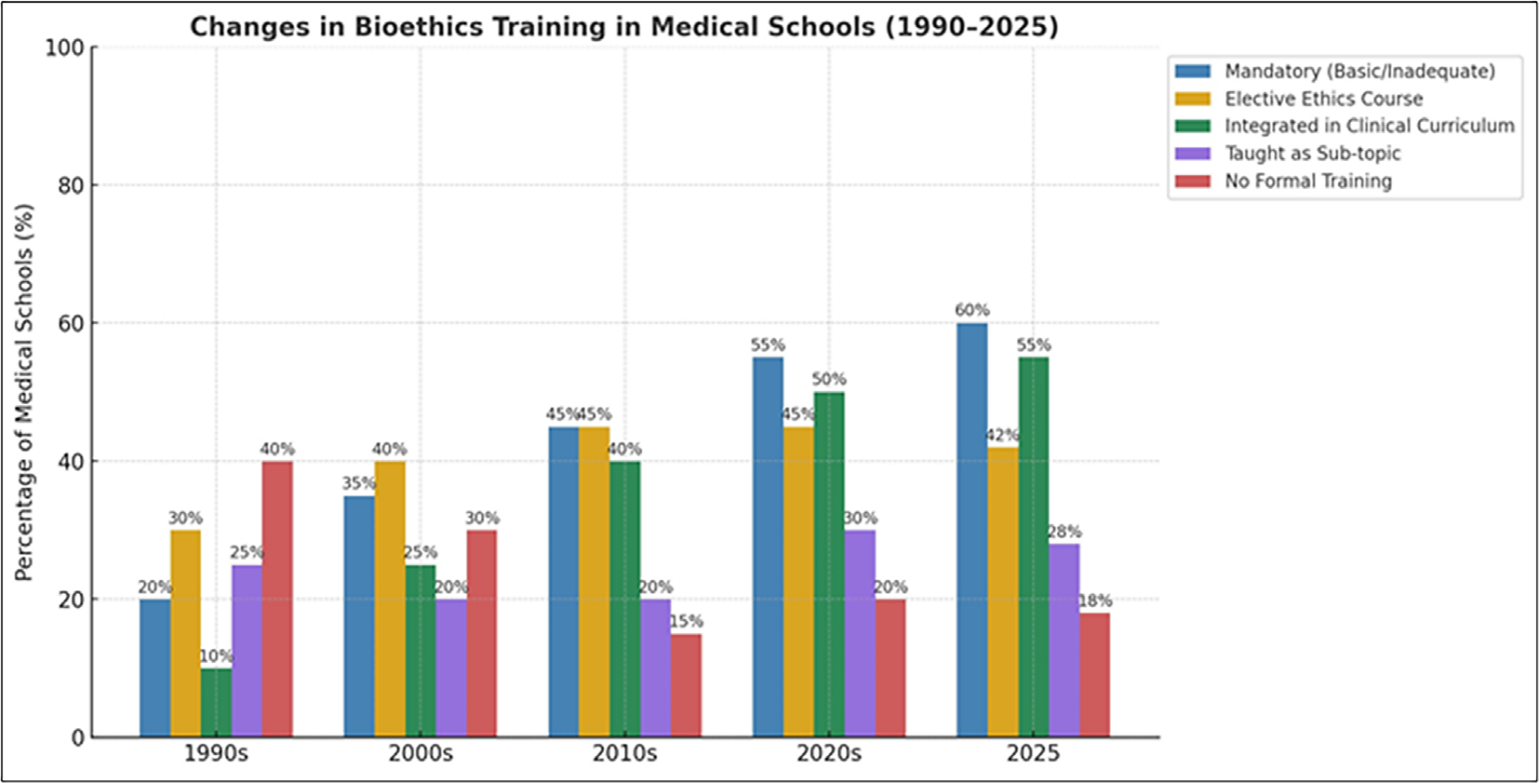
The evolution of bioethics education across global medical schools over the past four decades and the current year. The data reflect trends in how ethics instruction is delivered, including basic mandatory coursework, elective courses, integration within the clinical curriculum, treatment as a sub-topic, and absence of formal training. While there has been a marked increase in both mandatory and integrated bioethics instruction, a significant number of institutions still offer only partial or no structured training. These trends underscore the growing recognition of ethics in medical education, while also highlighting ongoing gaps in its practical application and consistency.

Unfortunately, most medical schools do not equip students to deal with such complicated situations and painful announcements [27]. Hopefully, this norm could change as soon as university administrations elucidate the necessity for such types of knowledge and skills for medical professionals. This gap in training is reflected in global trends over the last several decades (Figure 3) [28–31]. Part of the solution could be the addition of mandatory classes such as bioethics and communication skills courses in the curricula of any health professional’s major. Alternatively, postgraduate programs in every medical school in this specific field could enrich the health professionals’ view and eventually improve physicians’ interaction with patients [32]. Additionally, counseling should officially be conducted by specialists. A patient with a fatal disease needs to be healed psychologically as well as physically. Multidisciplinary institutional advisory boards may help to build up common rules for the handling of terminal patients, taking into account the family’s and patients’ individuality. Moreover, local supportive teams, composed of scientists and disease survivors, could be established in order to provide financial and psychological support to groups of patients with progressive or fatal diseases. One of the most highly valued challenges is for patients to continue to feel members of the community and to realize that other people have faced similar situations and have survived [33].

It is our firm belief that physicians need to look at the situation from the point of view of the patient. Equality and honesty should exist in the patient-physician relationship. Through that, the patient will manage to maintain their dignity and self-esteem. Simultaneously, they will achieve better compliance with the instructions of their therapists. Truth is the only way for a patient to move on to the stage of acceptance and finally be able to live his truth and own life selections as long as he can.

## Epilogue

Truth seems to remain the only way for a patient to deal with his disease, and telling the truth in critically ill patients is accompanied by the concept of personalized medicine and care. Definitely, the quality that truth assumes when presented strongly depends on the physician’s expertise, levels of communication, ability to psychologically analyze each individual patient, and the social and cultural background of the patients. These requirements emphasize the necessity of more focused and adequate training of the healthcare professionals, as well as further scientific research in the field. This path will hopefully manage to alleviate the feeling of abandonment and ignorance in the patient-physician relationship. Explanation to patients is the key to a successful outcome for all parties.

## Data Availability

Data on the literature search performed for this study are available upon request.

## References

[1] Meggiolaro E, Berardi MA, Andritsch E, Nanni MG, Sirgo A, Samorì E, Farkas C, Ruffilli F, Caruso R, Bellé M, Juan Linares E, de Padova S, Grassi L (2016) Cancer patients’ emotional distress, coping styles and perception of doctor-patient interaction in European cancer settings. Palliat Support Care 14(3), 204–211. 26155817 10.1017/S1478951515000760

[2] Landen CN Jr, Younger NO, Collins Sharp BA, Underwood PB (2003) Cancer patients’ satisfaction with physicians: Princess Margaret Hospital Satisfaction with Doctor Questionnaire results. Am J Obstet Gynecol 188(5), 1177–1179. 12748470 10.1067/mob.2003.281

[3] Wang JH, Adams IF, Tucker-Seeley R, Gomez SL, Allen L, Huang E, Wang Y, Pasick RJ (2013) A mixed method exploration of survivorship among Chinese American and non-Hispanic White breast cancer survivors: the role of socioeconomic well-being. Qual Life Res 22(10), 2709–2720. 23591710 10.1007/s11136-013-0374-0 PMC3855903

[4] Takayama T, Yamazaki Y, Katsumata N (2001) Relationship between outpatients’ perceptions of physicians’ communication styles and patients’ anxiety levels in a Japanese oncology setting. Soc Sci Med 53(10), 1335–1350. 11676404 10.1016/s0277-9536(00)00413-5

[5] Bregman L (1989) Dying: a universal human experience? J Relig Health 28(1), 58–69. 24276751 10.1007/BF00987503

[6] Kübler-Ross E (1969) On death and dying. New York, Macmillan.

[7] Kübler-Ross E, Kessler D (2005) On grief and grieving: finding the meaning of grief through the five stages of loss. New York, Scribner.

[8] Telford K, Kralik D, Koch T (2006) Acceptance and denial: implications for people adapting to chronic illness: literature review. J Adv Nurs 55(4), 457–464. 16866841 10.1111/j.1365-2648.2006.03942.x

[9] Laxmi S, Khan JA (2013) Does the cancer patient want to know? Results from a study in an Indian tertiary cancer center. South Asian J Cancer 2(2), 57–61. 24455553 10.4103/2278-330X.110487 PMC3876664

[10] Tan MS, Narasimhalu K, Ong SY (2012) Letting the cat out of the bag: shifting practices of cancer disclosure in Singapore. Singapore Med J 53(5), 344–348. 22584976

[11] Samimi Ardestani SM, Faridhosseini F, Shirkhani F, Karamad A, Farid L, Fayyazi Bordbar MR, Motlagh A (2015) Do cancer patients prefer to know the diagnosis? A descriptive study among Iranian patients. Iran J Psychiatry Behav Sci 9(4), e1792. 26834800 10.17795/ijpbs-1792 PMC4733304

[12] Pronzato P, Bertelli G, Losardo P, Landucci M (1994) What do advanced cancer patients know of their disease? A report from Italy. Support Care Cancer 2(4), 242–244. 7522106 10.1007/BF00365729

[13] Gazielly DF, Scarlat MM (2021) Back to basics. The value of clinical examination and traditional human contact between a patient and his physician compared with procedural standardized virtual or presential consultation: a narration. Int Orthop 45(6), 1387–1389. 34032914 10.1007/s00264-021-05086-2

[14] Sioutis S, Reppas L, Bekos A, Limneos P, Saranteas T, Mavrogenis AF (2021) The Hippocratic Oath: analysis and contemporary meaning. Orthopedics 44(5), 264–272. 34590941 10.3928/01477447-20210819-08

[15] Quaile A, Mavrogenis AF, Scarlat MM (2024) What happened to “bedside manner”? Int Orthop 48(4), 885–887. 38353708 10.1007/s00264-024-06112-9

[16] Foissey C, Fauvernier M, Fary C, Servien E, Lustig S, Batailler C (2020) Total hip arthroplasty performed by direct anterior approach – does experience influence the learning curve? SICOT J 6, 15. 32500856 10.1051/sicotj/2020015 PMC7273835

[17] Kouyoumdjian P, Mansour J, Assi C, Caton J, Lustig S, Coulomb R (2020) Current concepts in robotic total hip arthroplasty. SICOT J 6, 45. 33258445 10.1051/sicotj/2020041 PMC7705325

[18] Anderson PM, Vollmann P, Weißenberger M, Rudert M (2022) Total hip arthroplasty in geriatric patients – a single-center experience. SICOT J 8, 12. 35380534 10.1051/sicotj/2022011 PMC8982179

[19] Derreveaux V, Schmidt A, Shatrov J, Sappey-Marinier E, Batailler C, Servien E, Lustig S (2022) Combined procedures with unicompartmental knee arthroplasty: high risk of stiffness but promising concept in selected indications. SICOT J 8, 4. 35191830 10.1051/sicotj/2022002 PMC8862640

[20] Ghersi A, Mansour J, Marchand P, Alrubaie A, Kouyoumdjian P, Coulomb R (2022) Surgical videos on the internet: is this a reliable pedagogical tool in residency training? SICOT J 8, 39. 36149275 10.1051/sicotj/2022039 PMC9503426

[21] Foissey C, Pineda T, Servien E, Fontalis A, Batailler C, Lustig S (2024) Adapting hip arthroplasty practices during the COVID-19 pandemic: assessing the impact of outpatient care sudden increase on early complications and clinical outcomes. SICOT J 10, 1. 38193980 10.1051/sicotj/2023037 PMC10775906

[22] Quaile A, Mavrogenis A, Scarlat M (2021) Managing patients’ expectations in orthopaedics. Int Orthop 45(3), 539–541. 33523242 10.1007/s00264-021-04952-3

[23] O’Hara NN, Kringos DS, Slobogean GP, Degani Y, Klazinga NS (2021) Patients place more of an emphasis on physical recovery than return to work or financial recovery. Clin Orthop Relat Res 479(6), 1333–1343. 33239518 10.1097/CORR.0000000000001583 PMC8133069

[24] Tagliaferri SD, Miller CT, Owen PJ, Mitchell UH, Brisby H (2020) Domains of chronic low back pain and assessing treatment effectiveness. Pain Pract 20(2), 211–225. 31610090 10.1111/papr.12846

[25] Lee WA, Lee WE, Law S, Lau WA, Leung S (2013) Managing psychosocial contributors in low back pain patients – a randomized controlled trial. J Orthop Trauma Rehab 17(1), 46–51.

[26] Wolfensberger A, Vuistiner P, Konzelmann M, Plomb-Holmes C, Leger B (2016) Clinician and patient-reported outcomes are associated with psychological factors in patients with chronic shoulder pain. Clin Orthop Relat Res 474(9), 2030–2039. 27357692 10.1007/s11999-016-4894-0 PMC4965376

[27] Searight HR, Gafford J (2005) Cultural diversity at the end of life: issues and guidelines for family physicians. Am Fam Physician 71(3), 515–522. 15712625

[28] Eckles RE, Meslin EM, Gaffney M, Helft PR (2005) Medical ethics education: where are we? Where should we be going? A review. Acad Med 80(12), 1143–1152. 16306292 10.1097/00001888-200512000-00020

[29] Alkabba AF, Hussein GM, Kasule OH, Jarallah J, Alrukban M, Alrashid A (2013) Teaching and evaluation methods of medical ethics in the Saudi public medical colleges: cross-sectional questionnaire study. BMC Med Educ 13, 122. 24020917 10.1186/1472-6920-13-122 PMC3850889

[30] Pergert P, Lützén K (2012) Balancing truth-telling in the preservation of hope: a relational ethics approach. Nurs Ethics 19(1), 21–29. 22140184 10.1177/0969733011418551

[31] Edwards S (2014) Telling the truth? Nurs Ethics 21(4), 383–384. 24842986 10.1177/0969733014526966

[32] Madhiwalla N (2013) The ethics of truth telling. South Asian J Cancer 2(2), 53–54. 24455547 10.4103/2278-330X.110472 PMC3876651

[33] Ong WY, Yee CM, Lee A (2012) Ethical dilemmas in the care of cancer patients near the end of life. Singapore Med J 53(1), 11–16. 22252176

